# Helicase-Like Transcription Factor (Hltf) Regulates G2/M Transition, Wt1/Gata4/Hif-1a Cardiac Transcription Networks, and Collagen Biogenesis

**DOI:** 10.1371/journal.pone.0080461

**Published:** 2013-11-20

**Authors:** Rebecca A. Helmer, Raul Martínez-Zaguilán, Janet S. Dertien, Candra Fulford, Oded Foreman, Vasum Peiris, Beverly S. Chilton

**Affiliations:** 1 Department of Cell Biology & Biochemistry, Texas Tech University Health Sciences Center, Lubbock, Texas, United States of America; 2 Department of Cell Physiology & Molecular Biophysics, Texas Tech University Health Sciences Center, Lubbock, Texas, United States of America; 3 Department of Pharmacology & Neuroscience, Texas Tech University Health Sciences Center, Lubbock, Texas, United States of America; 4 The Jackson Laboratory, Sacramento, California, United States of America; 5 Department of Pediatrics, Texas Tech University Health Sciences Center, Lubbock, Texas, United States of America; Beijing Institute of Microbiology and Epidemiology, China

## Abstract

HLTF/Hltf regulates transcription, remodels chromatin, and coordinates DNA damage repair. Hltf is expressed in mouse brain and heart during embryonic and postnatal development. Silencing Hltf is semilethal. Seventy-four percent of congenic C57BL/6J Hltf knockout mice died, 75% within 12-24 hours of birth. Previous studies in neonatal (6-8 hour *postpartum*) brain revealed silencing Hltf disrupted cell cycle progression, and attenuated DNA damage repair. An RNA-Seq snapshot of neonatal heart transcriptome showed 1,536 of 20,000 total transcripts were altered (p < 0.05) - 10 up- and 1,526 downregulated. Pathway enrichment analysis with MetaCore™ showed Hltf’s regulation of the G2/M transition (p=9.726E^-15^) of the cell cycle in heart is nearly identical to its role in brain. In addition, Brca1 and 12 members of the Brca1 associated genome surveillance complex are also downregulated. Activation of caspase 3 coincides with transcriptional repression of Bcl-2. Hltf loss caused downregulation of Wt1/Gata4/Hif-1a signaling cascades as well as Myh7b/miR499 transcription. Hltf-specific binding to promoters and/or regulatory regions of these genes was authenticated by ChIP-PCR. Hif-1a targets for prolyl (P4ha1, P4ha2) and lysyl (Plod2) collagen hydroxylation, PPIase enzymes (Ppid, Ppif, Ppil3) for collagen trimerization, and lysyl oxidase (Loxl2) for collagen-elastin crosslinking were downregulated. However, transcription of genes for collagens, fibronectin, Mmps and their inhibitors (Timps) was unaffected. The collective downregulation of genes whose protein products control collagen biogenesis caused disorganization of the interstitial and perivascular myocardial collagen fibrillar network as viewed with picrosirius red-staining, and authenticated with spectral imaging. Wavy collagen bundles in control hearts contrasted with collagen fibers that were thin, short and disorganized in Hltf null hearts. Collagen bundles in Hltf null hearts were tangled and fragmented. Thus, silencing Hltf during heart organogenesis compromised DNA double-strand break repair, and caused aberrant collagen biogenesis altering the structural network that transmits cardiomyocyte force into muscle contraction.

## Introduction

Congenital heart defects (CHDs) - 1 in 100 newborns - result from errors in the development of heart valves, chambers and blood vessels [1]. Although present at birth, CHDs vary in severity and often emerge as complications of aging [2]. Growth and development of the mammalian heart occurs in embryonic and postnatal stages. Transgenic and knockout mouse models have helped to characterize transcription factors (MEF2, GATA4, SRF, NFkB, and NFAT) that regulate the fetal cardiac gene program [3]. These transcription factors also participate in the reactivation of the fetal cardiac gene program in response to physiological stress. However, the framework of the entire gene program is still under construction. For example, little is known of the molecular mechanism(s) that regulate [4-6] the formation of coronary vessels and the fibrous extracellular matrix (ECM). In mouse, the proepicardium (PE) forms on E9.25, and the proepicardium-derived epicardium envelopes the myocardium by E10.5. Select epicardium-derived cells (EPDCs) undergo an epithelial-mesenchymal transition (EMT), and invade the subepicardial space and myocardium. EPDCs differentiate into a variety of cell types including cardiac fibroblasts that are responsible for synthesis of ECM, a scaffold for fibroblast-myocyte-capillary interactions [6]. The matrix, which is composed of collagen, elastin and fibronectin, physically connects myocytes, prevents slippage between cells, and aligns bundles of myocytes to coordinate transmission of force in myocardium function. Although the inductive signals that initiate formation of the PE are not well characterized, synergy between Gata4 and Wilms’ tumor (Wt1) proteins is mandatory [7-9]. Wt1 is required for PE development, and loss of Gata4 prevents PE outgrowth. Our recent work with a helicase-like transcription factor (Hltf) null mouse model indicates Hltf has an important role in cardiac morphogenesis through its regulation of Gata4 and Wt1. Hltf also regulates homeostasis of the three-dimensional ECM collagen scaffold via its transcriptional control of Hif-1a.

HLTF/Hltf was first described as a DNA-binding protein [10-12] and subsequently authenticated as a transcription factor [13-21]. As a SWI/SNF family member, HLTF remodels chromatin architecture [22-24]. HLTF also participates in DNA damage repair, and tumor suppression [25]. In mice, Hltf is expressed in heart as early as E8.5-9.5 [12,26]. Thereafter, transcripts accumulate in heart and brain [12]. To define HLTF’s function we generated Hltf null mice [27]. Initially, 64% of the Hltf null mice died, 48% within 12-24 hours of birth. Once congenic on the C57BL/6J background, 74% of Hltf null mice died, 75% within 12-24 hours of birth. Comparative transcriptomic (RNA-seq) analyses of neonatal (6-8 hour *postpartum*) hearts and brains revealed the Hltf null phenotype derives from defects in the G2/M transition (p=3.360E^-10^). This finding is consistent with Hltf’s role in the maintenance of genomic stability. Additional findings unique to Hltf null heart – decreased transcripts for Wt1 and Gata4 and their downstream targets, downregulation of transcripts for Brca1/BASC complex in DNA damage repair, disorganization of the collagen fibrillar network via decreased transcription of hypoxia-inducible factor (Hif-1a) and its downstream targets, and decreased transcripts for Myh7b/miR499 – demonstrate a critical role for Hltf in heart function.

## Materials and Methods

### Reagents and Kits

Santa Cruz Biotechnology, Inc. (Santa Cruz, CA) was the source of antibodies to N-terminal residues of HLTF (sc-27542X), and Protein A/G PLUS-Agarose (sc-2003) beads. Abcam (Cambridge, MA) was the source of antibodies to Hltf residues 600-700 (ab17984) and the Caspase 3 Assay Kit (ab39401). GE HealthCare (Pittsburgh, PA; formerly Amersham Biosciences) was the source of donkey anti-rabbit IgG HRP (NA934V). Ready Gel Tris-HCl precast polyacrylamide gels (7.5%, #161-1154), Kaleidoscope Prestained Standards (161-0324) and Immuno-Star Western Kit (170-5070) were purchased from Bio-Rad (Hercules, CA). Magic Mark XP (LC5602) western protein standards were purchased from the Invitrogen division of Life Technologies (Grand Island, NY). PerkinElmer Life Science Products (Waltham, MA) was the source of Kodak Film (NEF596). OneTouch Ultra Mini and OneTouch Ultra Mini Blue test strips were purchased from LifeScan (Malpitas, CA), a Johnson & Johnson Company, for blood glucose monitoring. For collagen detection, ScyTek Laboratories, Inc. (Logan, UT) was the source of the Picrosirius red stain kit (PSR-2), and Chondrex, Inc, (Redmond, WA) was the source of the Hydroxyproline assay kit (#6017). 

Mouse heart extract was prepared with the Active Motif (Carlsbad, CA) kit (#40010). DNeasy Blood & Tissue Kit (69506) was purchased from Qiagen (Valencia, CA) for DNA purification. Expand Long Template PCR System Buffer (11681842001) and PCR nucleotide mix (11814362001) were purchased from Roche Applied Science (Indianapolis, IN). NucleoSpin RNA II, a complete kit for isolation and purification of total RNA was purchased from Clontech (Mountain View, CA). SuperScript III first-strand synthesis system for RT-PCR was purchased from the Invitrogen division of Life Technologies (Grand Island, NY). PCR primers (Table 1) were synthesized by Midland Certified Reagent Company (Midland, TX). MetaPhor® agarose (50181) was purchased from Lonza Rockland, Inc. (Rockland, ME). Promega (Madison, WI) was the source of agarose gel markers φX174 (G176A), and a 50-bp ladder (G4521). For RNA-seq, Otogenetics Corporation (Norcross, GA) used the following reagents: Ribo-Zero rRNA Removal Kit from Epicentre (Madison, WI), an Illumina company; NEBNext mRNA Sample Prep kit (E6110) and NEBNext reagents (E6040) from New England Biolabs (Ipswich, MA).

**Table 1:** PCR Primers.

Name	Sequence
Promoter Gata4 Forward	5΄-CCGCAAGGACGTCGGGCTGCACTG-3΄
Promoter Gata4 Reverse	5΄-GCTCCCGGCGCGGTTCCCC-3΄
Promoter Myh7b Forward	5΄-CTGAGTGACTGCCTCTCTCCGCCTTTGTG-3΄
Promoter Myh7b Reverse	5΄-CTGGCACCACGGTGAGGACAGGG-3΄
Exon 7 Myh7b Forward	5΄-CTACACATACTCGGGCCTCTTCTGTGTCACC-3΄
Exon 7 Myh7b Reverse	5΄-GTTGTCGTTCCTCAGAGTCTTGGCGTTGCC-3΄
Promoter Hif1a Forward	5΄-GATTCAAGTGGTCTTCCTGCTTCAGC-3΄
Promoter Hif1a Reverse	5΄-GGGACTCATCCCAGGCGGG-3΄

### Hltf Null Mice

Hltf knockout mice were developed in collaboration with genOway (Lyon, France) as previously documented [27]. Conventional wisdom that N3 mice have a 129v/C57BL/6J genetic background of 12.5%:87.5% was combined with marker-assisted breeding (speed congenics). Six Hltf heterozygous males were tested with a 104-marker panel that distinguished between C57BL/6J and 129Sv. Results ranged from 74.5-87.4% with an average of 81.8% C57BL/6J. Two males, one at 87.4% C57BL and the other at 85.4% C57BL were used for the next generation of backcrossing. All studies with the Hltf-deficient mouse strain – backcrossed into a C57BL/6J congenic background for 10 generations – were conducted in accord with the NIH Guidelines for the Care and Use of Laboratory Animals, as reviewed and approved by the Animal Care and Use Committee at Texas Tech University Health Sciences Center [NIH Assurance of Compliance A3056-01; USDA Certification 74-R-0050, Customer 1481]. TTUHSC's IACUC specifically approved this study. All efforts were made to minimize pain and suffering.

The RT component of RT-PCR was performed according to the manufacturer’s instructions for the combined use of oligo-dT and random hexamer priming. Each 50µl PCR reaction consisted of template DNA, primer pairs (15 pmol each, Table 1), dNTPs (0.5 mM), reaction buffer 3 (0.1 Vol), and expand long template polymerase (2.6 U). Reaction conditions for the knockout allele were previously described [26]. A five-step touchdown PCR was performed for ChIP as follows: 120 s at 94°C, followed by 5 cycles 94°C, 30 s; 69.5°C, 30 s; 68°C, 120 s; 5 cycles of 94°C, 30 s; 69°C, 30 s; 68°C, 120 s; 5 cycles of 94°C, 30 s; 68.5°C, 30 s; 68°C, 120 s; 5 cycles of 94°C, 30 s; 68°C, 30 s; 68°C, 120 s; 15 cycles of 94°C, 30 s; 67.5°C, 30 s; 68°C, 120 s; and a final extension for 8 min at 68°C. Reaction conditions for the PCR component of RT-PCR were as follows: 120 sec at 94 C, followed by 35 cycles of 94 C for 30 sec, 65 C for 30 sec, and 68 C for 120 sec, and a final extension for 480 sec at 68 C. At the conclusion of each reaction, samples were rapidly cooled to 4 C, and amplicons were resolved/visualized by MetaPhor® agarose gel electrophoresis, subcloned and sequenced.

### Techniques

Microscopy and chromatin immunoprecipitation-PCR (ChIP-PCR) were performed as previously detailed [16,19,20,28,29]. For histological evaluation, hearts were removed from newborn mice at 6-8 hours of age, and emersion-fixed in a variety of formalin-based fixatives. Hearts were paraffin embedded and serially sectioned (5-8 μm). Hematoxylin and eosin (H&E) staining was performed by TTUHSC Department of Pathology. Picrosirius red stain kit and the hydroxyproline assay kit were used according to the manufacturer’s protocol. Hearts from null and wildtype mice were batch stained together. Stained sections were evaluated by light microscopy.

Differential interference contrast (DIC) images (63x oil) for picrosirius red with excitation and barrier filters for FITC (intrinsic cytoplasmic and elastic fiber green fluorescence) were captured with a Zeiss AxioVert 200 inverted fluorescence microscope, AxioVision software, and an AxioCam-camera. OD values (530-560 nm) for the hydroxyproline content of standards and samples were read with the Infinite M100 PRO Quadruple monochromator microplate reader (Tecan). Intrinsic fluorescence of tissue, including elastic and collagen fibers, were obtained using spectral imaging [30] of unstained (deparaffinized) and picrosirius red-stained sections. To do this, we used an Olympus1X70 inverted microscope coupled to a Spectro-Pro-300i spectrograph via a C-mount adaptor (IX-TVAD). A cryogenically cooled (-100 C), charge-coupled device (CCD) camera [1,340 X 400-pixel imaging array (pixel 20 x 20 μm)] with an ST133 controller, and WinSpec/32 spectroscopic software (version 2.5.10.1) completed the imaging system. The grating in the spectrophotometer was blazed at 500 nm with slit widths at 3, 1, and 0.5 μm. The length of the cell regions demarcated by the 0.5-μm slit was subdivided into discrete regions (20 pixels each) allowing the acquisition of images with high temporal, spectral, and spatial resolution. Spectral imaging utilized 488-nm excitation and 505 dichroic filters. A 530-nm emission filter was added for imaging. Fluorescence data were converted to ASCII format prior to analysis with SigmaPlot (version 8.0). 

### Fetal Echocardiology

VisualSonic cardiac echocardiography was performed on pups *in utero* in term pregnant females (gestation days 19-21) at the Mouse Cardiovascular Phenotyping Core (MCPC), Washington University School of Medicine in St. Louis. Full echocardiograms of each fetal heart included: two-dimensional short- and long-axis cineloops of the heart from the parasternal view; two-dimensional guided M-mode image of the left ventricle and basal structures; Doppler recordings of trans-mitral, aortic, and pulmonary valve velocities; Doppler tissue recordings of mitral annular and left ventricular segmental wall motion velocities. Females and pups were euthanized at the end of the study. 

### RNA-seq

Individual samples [6-8 hearts/sample x 3 biological replicates for test and control mice = 6 total samples] were flash frozen and sent to Otogenetics Corp. (Norcross, GA) for RNA-seq assays. Total RNA was isolated; its integrity and purity were assessed using Agilent Bioanalyzer and OD260/280 (Table 2). As previously described [27], each sample was rRNA-depleted using the appropriate Ribo-Zero rRNA Removal Kit according to the manufacturer. NEBNext mRNA Sample Prep kit was used to generate cDNA from the rRNA-depleted RNA. The cDNA was profiled using Agilent Bioanalyzer, and subjected to Illumina library preparation using NEBNext reagents. The quality, quantity and size distribution of the Illumina libraries were determined using an Agilent Bioanalyzer 2100. The libraries were then submitted for Illumina HiSeq2000 sequencing according to standard operation. Paired-end 100 nucleotide reads were aligned to genomic assembly mm9 (Table 2) and analyzed using the platform provided by DNAnexus, Inc. (Mountain View, CA).

**Table 2:** Sample quality control and RNA-seq outcome.

Sample ID	OD260/280	RIN**^*†*^**	Total Bases	Total Reads	Mapped Reads
1-Control	2.14	9.1	2,866,775,400	28,667,754	37.45%
2-Control	2.08	8.8	3,554,260,200	35,542,602	70.05%
3-Control	2.13	9.2	3,075,605,600	30,756,056	66.74%
4-Null	2.14	9.8	3,579,700,400	35,797,004	28.34%
5-Null	2.08	9.5	3,317,503,200	33,175,032	33.81%
6-Null	2.12	9.4	4,029,281,000	40,292,810	40.31%

† High RNA integrity number (RIN) scores (7-10) and a narrow distribution of scores (1-1.5)

from an Agilent Bioanalyzer indicated high RNA sample quality.

### Putative Hltf-binding sites

We previously identified a consensus Hltf (C/A C T/A T A/T/G T/G) binding site [16]. Genomatix (MatInspector) used our data to develop a proprietary algorithm for the identification of putative Hltf binding sites [31]. To date, three investigators [22-24] successfully used the algorithm. The algorithm aided the identification of putative Hltf binding sites in the regulatory/promoter regions of the Gata4, Hif-1a and Myh7b/miR499 genes.

### Data Analysis

RNA-seq in conjunction with 3SEQ/transcriptome was used to quantify expression levels by comparing sequencing reads against a reference transcriptome. Each transcript was quantified by calculating its RPKM (reads per kilobase of transcript per million mapped reads) enabling direct comparisons of expression levels among transcripts and across experimental conditions. RPKM and total read counts were reported for each gene.

Otogenetics via DNAnexus provided an unbiased gene expression analysis report of RNA-seq; alternative splicing analysis of Hltf; mutation/RNA-editing analysis and parallel comparison of expression profiles between null and control samples. FPKM (fragments per kilobase of transcript per million mapped reads) were mapped against mm9 with Tophat (V2.0.5) and cufflinks.cuffdiff (V 2.0.2) for parallel analysis of six mouse RNA-seq samples. Differences between samples were considered statistically significant at q < 0.05. Using a free trial, data were imported to MetaCore™ for pathway analysis (GeneGO, Thomson Reuters, New York, NY). Standard enrichment parameters (1.1, p < 0.05) were used. Analyses from brain and heart were merged with MetaCore™. 

All statistical tests were conducted with GraphPad Prism V.6.0b (GraphPad Software). Caspase 3 assays were performed on whole heart extract according to the manufacturer’s protocol. Triplicate values were evaluated by ANCOVA (*p* < 0.05 significance level). The *p* value for the difference between elevations caused by the presence or absence of Hltf expression was *p* = 0.0001. Regression analyses of expression level were measured by RPKM for control vs. Hltf null values. Hydroxyproline assays were performed on HCl (12N) hydrolyzed whole hearts according to the manufacturer’s protocol. A standard curve with hydroxyproline standards was evaluated by linear regression (r=0.99941). Replicate values of serially diluted samples from null (n=3; 8-9 hearts/sample) and control (n=1; 8-9 hearts/sample) mice were compared by Student’s *t*-test (*p* < 0.05 significance level). Blood glucose levels were compared by the Mann-Whitney U-test (*p* < 0.05 significance level). The *p* value was *p*=0.0084.

## Results

**Figure 1:**
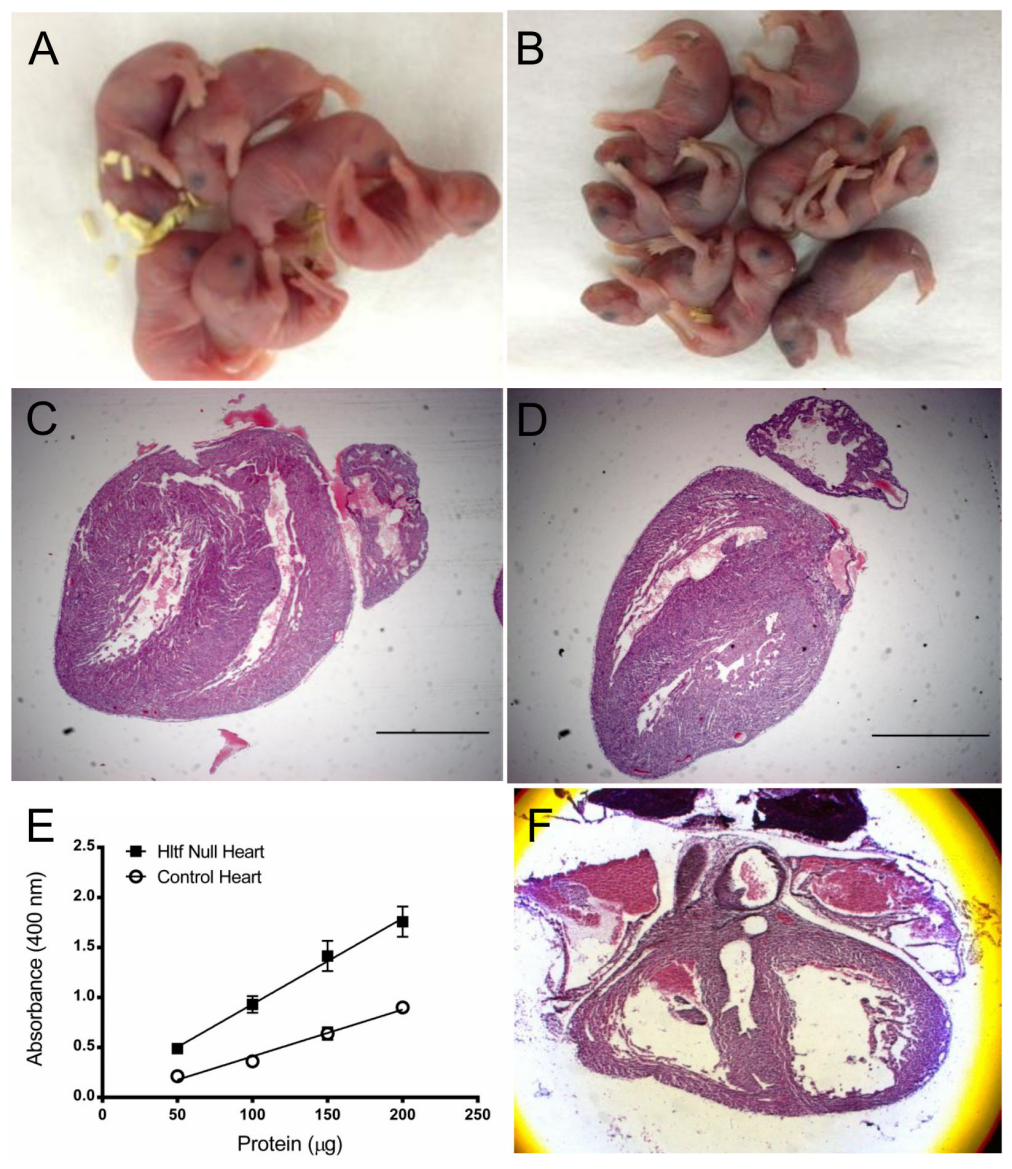
Hltf null phenotype compared with controls at 6-8 hours postpartum. Compared with controls (A), Hltf null pups developed cyanosis (B). Deviation in heart shape between control (C) and Hltf null mice (D) are evident in H & E stained sections. Increased (p<0.0001) active caspase 3 (E) showed elevated apoptosis in Hltf null hearts. One of five null pups had a rare left coronary artery fistula (F).

In collaboration with genOway, a Cre-lox strategy was used to generate constitutive Hltf null mice [27]. The resulting Hltf null mice have Hltf deleted from all tissues. Initially, 64% of Hltf null mice died, 48% within 12-24 hours of birth. Once congenic (N10) on the C57BL/6J background (n=732), 74% of Hltf null mice died, 75% within 12-24 hours of birth. At parturition, Hltf null mice and their littermate controls breathed freely and acquired a characteristic pink color suggesting normal lung and diaphragm function. Collectively, they displayed a suckling reflex, drank immediately after birth, and milk was visible in their stomachs. However, at 6-8 hours *postpartum* three of four null pups were hypoglycemic (Figure S1). At 12-24 hours *postpartum*, null pups developed progressive cyanosis (Figure 1A, B), with occasional gasping in some, and became moribund.

Because reduced cardiac output is one cause of peripheral cyanosis, we began a systematic characterization of Hltf null and control hearts 6-8 hours *postpartum*. As shown in Figure 1C, D, the shape of newborn Hltf null hearts (n=14) is more elongated (primitive) than controls (n=5). Biochemical (Figure 1E) evidence for increased apoptosis in Hltf null hearts supports a role for Hltf as a survival factor. When VisualSonic echocardiography was used to evaluate pups *in utero*, only one of five null pups displayed abnormal function (Video S1) concomitant with a left coronary artery fistula (Figure 1F). Because these findings do not explain the high neonatal death rate, the working hypothesis – newborn Hltf null mice die because their hearts are unable to meet the metabolic demands of extra-uterine life – was evaluated by gene expression profiling.

**Figure 2:**
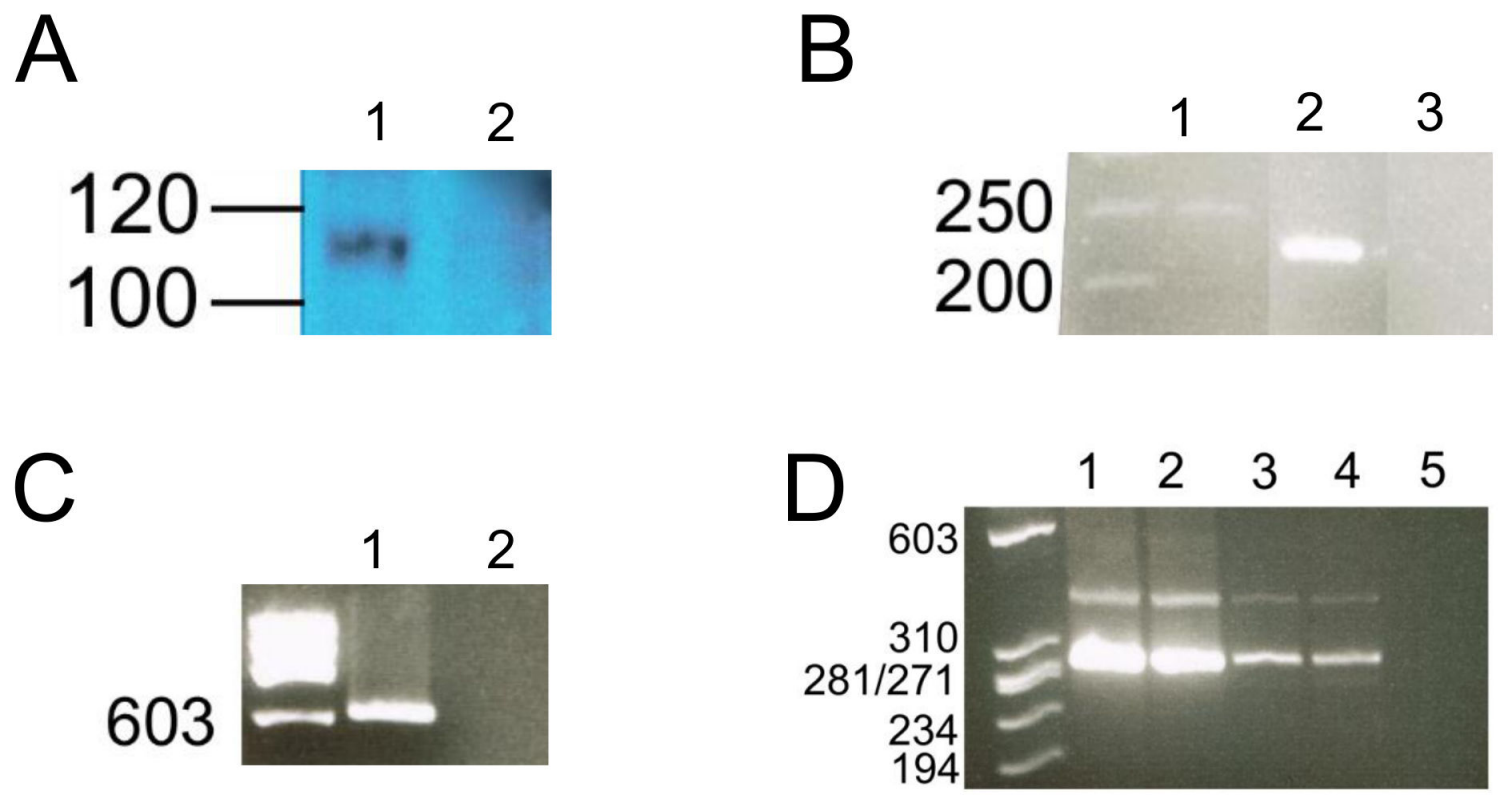
Western blot, ChIP-PCR and competitive RT-PCR. Panel A, Western blotting confirmed the presence of full-length Hltf protein (116-kDa between molecular markers 120 and 100 kDa) in hearts of control mice (lane 1), compared with the absence of protein expression in hearts of Hltf null mice (lane 2). Panel B, electrophoretic resolution of a single population of amplicons from touchdown PCR of ChIP confirmed Hltf bound the transcriptionally active regulatory regions of the promoters of Gata4 (248-bp; lane 1) and Myh7b/miR499 (228-bp; lane 2). Water blank control (lane 3) and molecular ladder (bp) are shown. Panel C, electrophoretic resolution of a single population of amplicons from touchdown PCR of ChIP confirmed Hltf bound the putative promoter of Hif-1a (600-bp, lane 1). Water blank control (lane 2) and φX174 markers are included. Panel D, analysis of competitive RT-PCR products by electrophoresis for the presence/absence of exon 7 in heart (lanes 1-2) and brain (lanes 3-4) revealed the ratio of amplicons without exon 7 (293-bp) to amplicons with exon 7 (390-bp) was the same in the presence (lane 1, 3) and absence (lane 2, 4) of Hltf. The water blank control (lane 5) and φX174 markers are added for completeness. The identity of each population of amplicons was verified by double-stranded sequencing.

Hltf is alternatively spliced, and little is known about the relative expression of message isoforms and their functional significance. DNAnexus alternative splicing analysis quantified the usage of each exon and each possible splice junction for Hltf in RNA-seq samples from Hltf control hearts (Table S1). Hltf isoform 1, the full-length splice variant (4955-bp), contains exons 1-25. Hltf isoform 2, the truncated splice variant (3059-bp), is comprised of exons 1-21 with exon 21 extended via a partial intron retention event. Quantification of isoform expression by Isoform FPKM tracking (cufflinks.cuffdiff) showed a 26:1 ratio for full-length isoform expression to the truncated splice variant. Only the 116-kDa Hltf protein derived from the full-length mRNA was detected by Western blot in heart extract (Figure 2A), suggesting the truncated isoform is lost to nonsense-mediated decay (NMD). Based on junction read counts, all additional splicing events are very low frequency, exon-skip events (Table S2).

**Figure 3:**
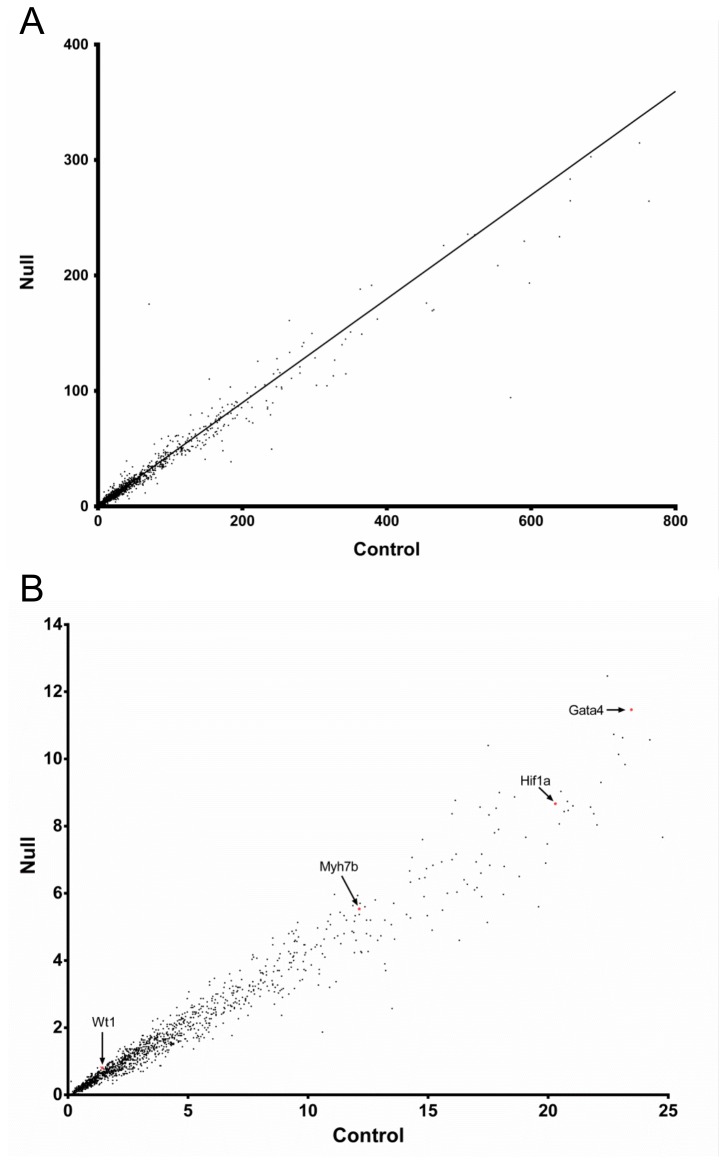
Scatter plot of expression level measured by RPKM: control vs. **Hltf knockout**. Panel A, each point is the mean of three replicate RPKM values for an individual gene in control heart plotted against values of the corresponding gene in Hltf null heart. The black line indicates the linear regression. R^2^ = 0.9818, correlation coefficient. Panel B, genes with statistically significant expression (each dot is the mean of three replicate RPKM values, differential expression >1.1 fold, and P < 0.05) in control vs. null heart samples. Genes with specific importance to this study [Gata4, Myh7b, Wt1, Hif-1a] are labeled with a red dot and a black arrow.

**Figure 4:**
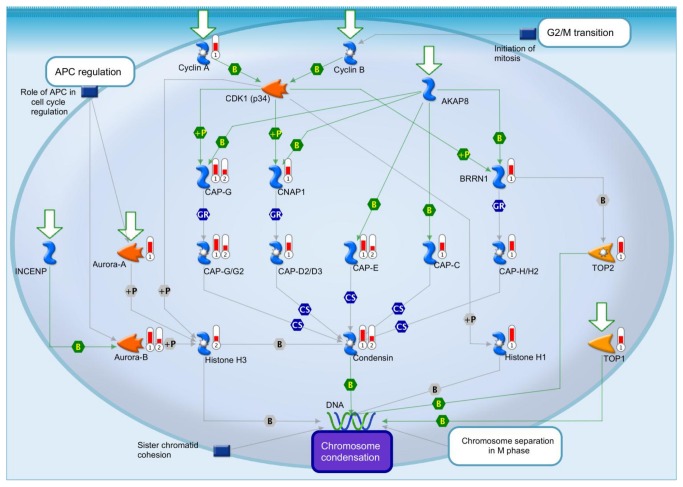
Hltf null hearts (1) and brains (2) share the same defects in the G2/M transition. Significant decreases (red thermometers = negative effects) in transcript expression of major components of the G2/M transition in Hltf null heart (1) and brain (2) are superimposed on the proprietary multicomponent canonical pathway map from the MetaCore™ database [Straight lines = interactions; Symbols = events; +P = phosphorylation; B = binding; GR = group relation; CS = complex subunit. Colors for lines and symbols are green for positive, and gray for unspecified].

Comprehensive analysis of the heart transcriptome (Figure 3A and Tables S3 and S4) showed 1,536 of 20,000 total transcripts were altered (p < 0.05) - 10 upregulated and 1526 downregulated - in Hltf null hearts (Figure 3B). MetaCore™ enrichment pathway analysis (Table 3) revealed Hltf is important in the regulation of cell cycle and DNA damage repair. We previously showed that Hltf’s most important role in brain is regulation of the G2/M transition of the cell cycle with an emphasis on transcript availability of major components in chromosome cohesion and condensation [27]. When new results from heart were superimposed on previous results from brain (Figure 4), it is clear that Hltf null hearts and brains share the same defects in the cell cycle. The phenotype of the Hltf null heart is further compromised by downregulation of the γ-tubulin gene (Tubg1) whose protein product is a critical component of the microtubule organizing center (MTOC), as well as downregulation of the Brca1gene and 12 members of the Brca1 associated genome surveillance complex (BASC) in DNA damage repair (Table 4). This collective loss of gene expression is expected to compromise DNA double-strand break repair, trigger pro-apoptosis signaling, and cause heart dysfunction.

**Table 3:** MetaCore™ enrichment pathway analysis.

Pathway	Category	pValue
Cell cycle	Chromosome condensation in prometaphase	9.726E-15
	Start of DNA replication in early S phase	9.821E-15
	The metaphase checkpoint	1.519E-13
	ATM/ATR regulation 0f G1/S checkpoint	1.980E-13
DNA damage	Role of Braca1 and Braca2 in DNA repair	1.703E-11
	Transition and termination of DNA replication	8.215E-11
Cell cycle	Regulation of G1/S transition (part 1)	1.099E-09
	Regulation of G1/S transition (part 2)	6.146E-09
	Sister chromatid cohesion	9.276E-09
	Nucleocytoplasmic transport of CDK/Cyclins	1.113E-08

**Table 4:** Downregulated (p<0.05) members of BASC.

Gene name	Fold-change	Function
Atm	2.45	Kinase
Atr	2.35	Kinase
Bard1	2.40	Binds Brca1 to stabilize both proteins
Fanca	3.82	Member, FA core complex
Fancg	2.00	Member, FA core complex
Fancl	2.26	Ubiquitin ligase
Fancd2	2.67	Check point arrest
MreII	2.56	Homologous recombination
Msh2	2.33	Homologous recombination
Rad51	2.38	S/G^2^ arrest
Rb1	2.70	Check point regulation
Stat1	2.30	Increases expression of interferon stimulated genes

The morphological phenotype of the Hltf null heart can be explained, in part, by decreased expression of Gata4 and Wt1 accompanied by reduced expression of genes that induce the EMT and promote angiogenesis (Table 5). Quantification of isoform expression by Isoform FPKM tracking (cufflinks.cuffdiff) identified only full-length isoforms for Gata4 and Wt1 in newborn mouse heart. Downregulation of transcripts for Wt1 is accompanied by downregulation of transcripts for its gene target, aldehyde dehydrogenase family 1, subfamily A2 (Aldh1a2, a.k.a. Raldh2), a marker gene used to identify proepicardial and epicardial cells. A role for Hltf in coordinating cardiac angiogenesis with cardiac growth is supported by regulation of transcripts for Gata4, Hif-1a, and Vegfa in Hltf null hearts. This is accompanied by downregulation of the glucose transporter Glut1, and the hexokinase Hk2, both of which are Hif-1a targets.

**Table 5:** Downregulated (p<0.05) contributors to the cardiac phenotype.

Gene name	Fold-change	Function
Actg1	1.76	Striations in cardiomyocytes
Adam12	1.90	Myoblast fusion
Adam17	2.16	Valvulogenesis
Adam19	2.23	Valvuloseptal morphogenesis
Adamts1	2.25	Stops trabeculation & compaction ventricular myocardium
Adamts9	2.22	Valvulogenesis
Cald1	2.07	Regulates smooth muscle contraction
Glut1 (Slc2a1)	2.28	Insulin-dependent glucose transporter
Hand1	4.19	Cardiac morphogenesis
Hk2	2.38	Catalyzes first step in glucose metabolism; antiapoptotic
Hif1a	2.34	Mediates cardioprotection
Icam1	2.26	Aortic valvulogenesis
Loxl2	2.45	Crosslinks elastin and collagen in ECM
Nfkb2	2.19	Induces angiogenesis; antiapoptotic
P4ha1	2.14	Required for collagen deposition
P4ha2	2.51	Required for collagen deposition
Plod2	2.43	ECM stiffening; collagen fiber alignment
Ppid	2.71	Accelerates collagen folding
Ppif	2.17	Accelerates collagen folding
Ppil3	2.57	Molecular chaperone in protein-folding
Prrx2	3.11	Coronary vascular morphogenesis
Snai1	1.92	Induces EMT
Snal2 (Slug)	1.81	Induces EMT; antiapoptotic
Vcam1	2.29	Cardiomyocyte differentiation; aortic valvulogenesis
Vcan	2.16	Interventricular septal formation
Vegfa	2.57	Induces angiogenesis and vasculogenesis; antiapoptotic
Wt1	1.79	Induces epicardial EMT
Zeb1	2.46	Induces EMT

**Figure 5:**
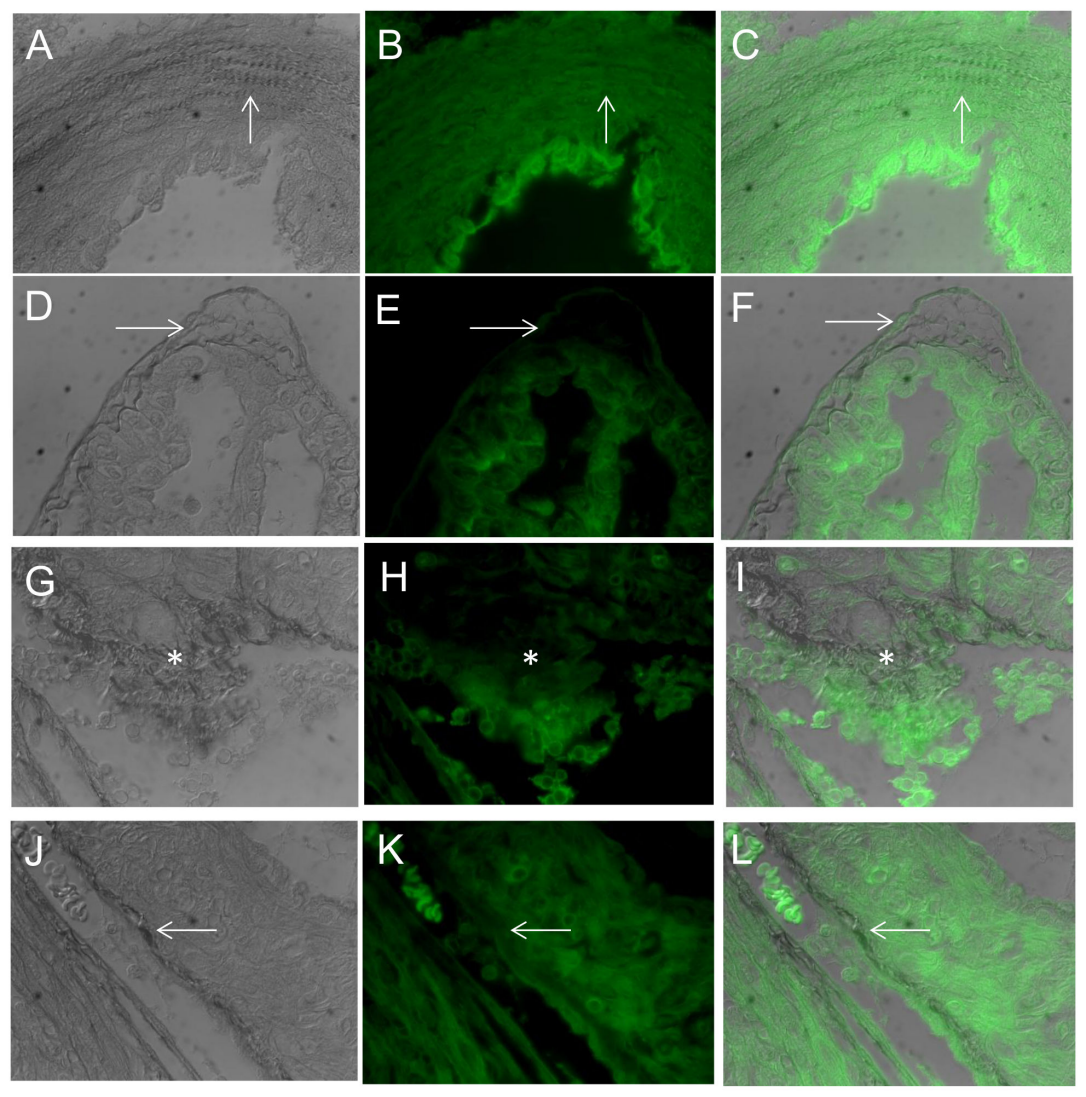
Photomicrographs of control (A-F) and Hltf null (G-L) hearts. Sections were stained with picrosirius red and imaged with transmitted DIC microscopy and epifluorescence. DIC images (left column) were merged with strong green (intrinsic) fluorescence of cells and elastic fibers (middle column) to show picrosirius red-stained collagen (right column). Note the wavy collagen bundles in the controls (C, F), compared with tangled bundles (I) and thin, short disorganized fibers (L) in Hltf null hearts. Arrows and asterisks (*) are used to show collagen lacks intrinsic fluorescence.

**Figure 6:**
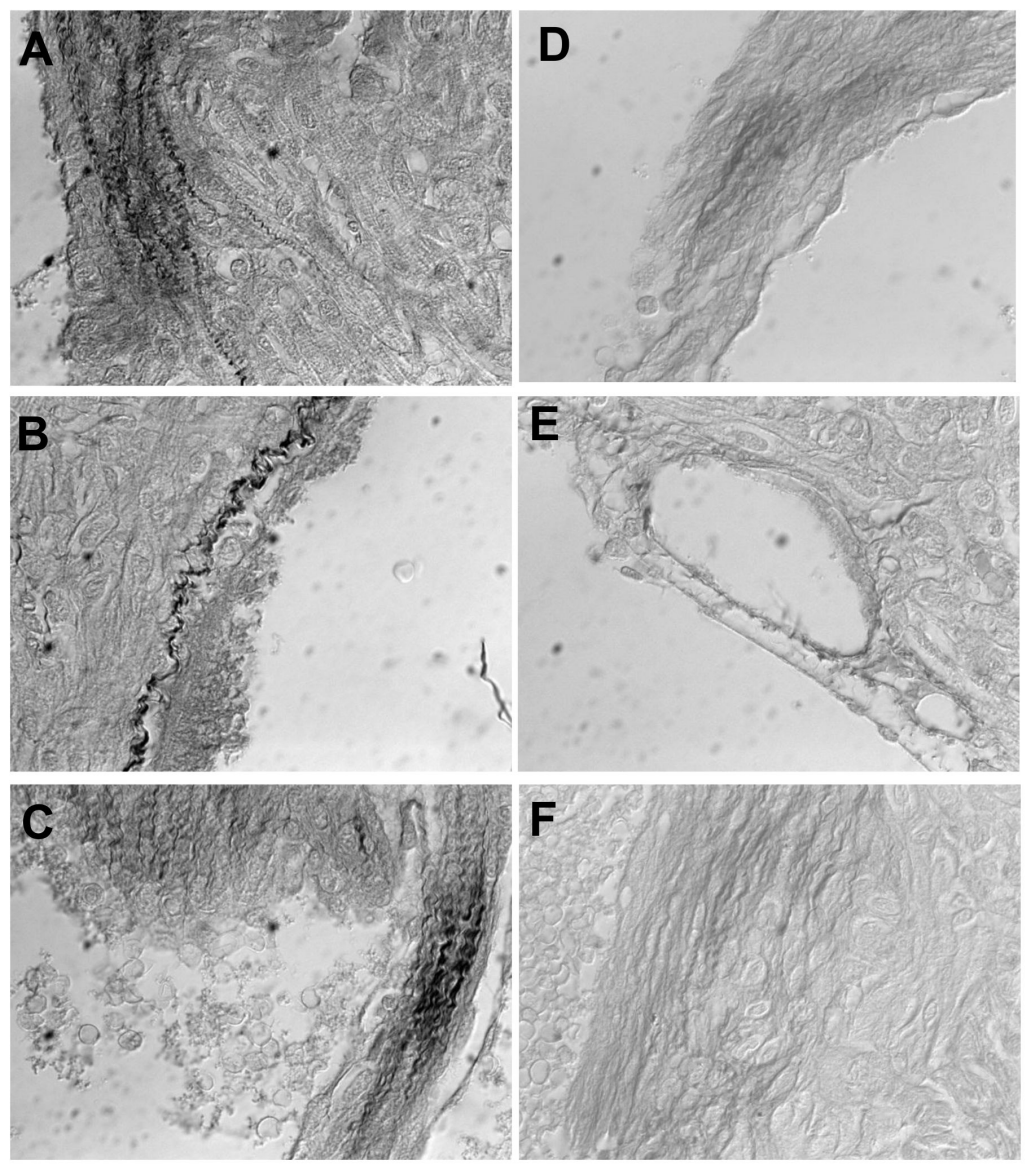
Photomicrographs (63X) of control (A-C) and Hltf null (D-F) hearts. Sections were stained with picrosirius red and imaged with transmitted DIC microscopy. Note the wavy interstitial (A, C) and perivascular (B) collagen in control hearts, compared with fragmented fibers (D, F) and thin, short fibers (D, E, F) in Hltf null hearts.

**Figure 7:**
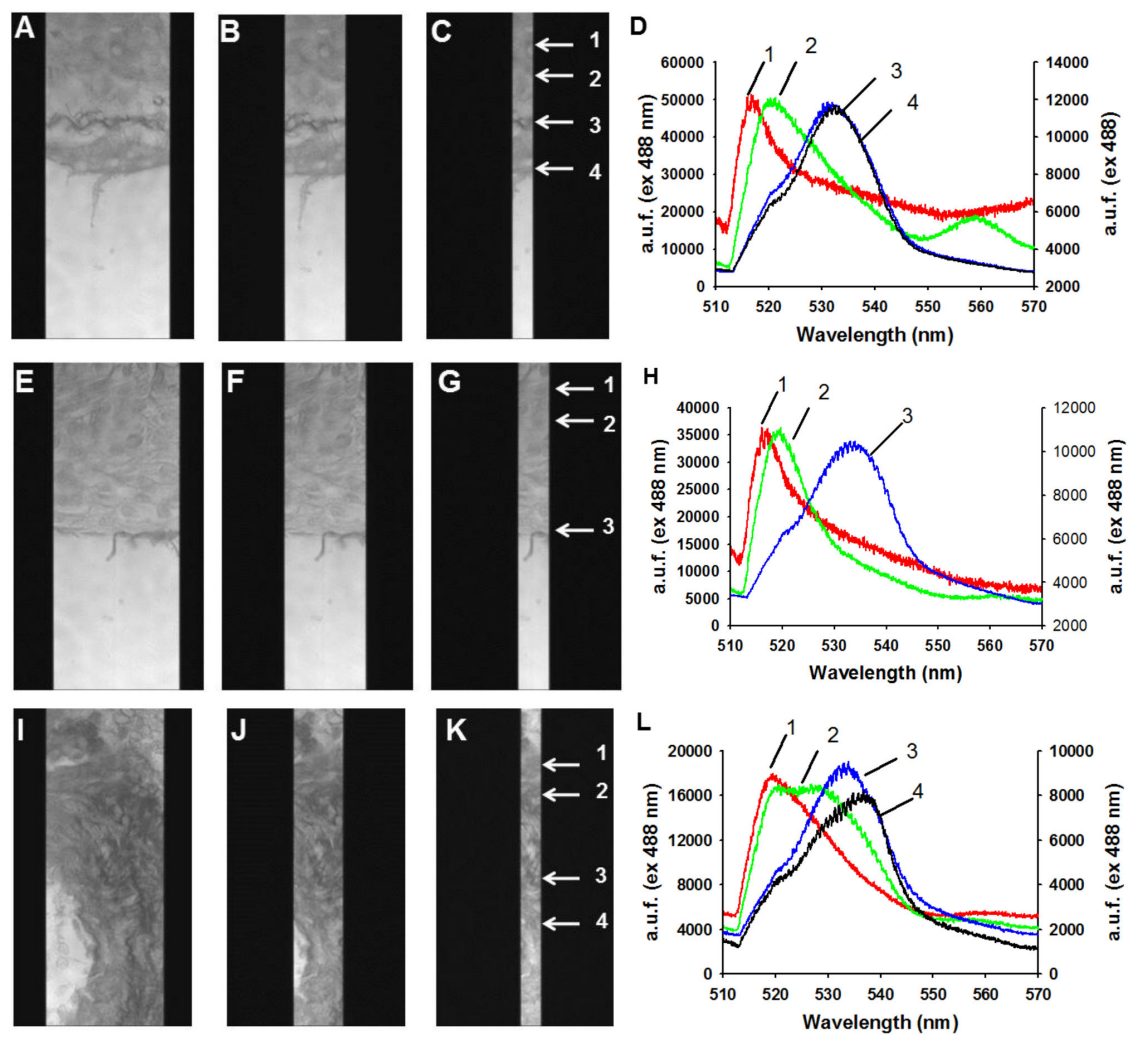
Spectral imaging microscopy of intrinsic fluorescence of control (A-D) and Hltf null (E-L) hearts. Heart sections stained with picrosirius red were imaged using 488 excitation (mercury lamp) and a halogen lamp to obtain zero order spectra of regions exhibiting both elastin and collagen. Cells were imaged using a 60x oil objective (N.A. 1.4) and a 500 ms exposure. Images A, E, and I were obtained using a 3 μm slit width on the spectrograph. For images B, F and J, the slit width was closed to 1 μm. For images C, G and K the slit width was closed to 0.25 μm. This ensured the highest spatial resolution from a discrete area. Notice in A-C the highly ordered structure of wavy collagen is apparent (arrows 3 and 4). The areas corresponding to elastin were obtained from regions of interest (ROI) at arrows 1 and 2. The corresponding first order spectra at 0.4 nm resolution (D) shows the ROI labeled as 1 and 2 correspond to elastin with peaks at 515 and 520 nm; whereas collagen (arrows 3 and 4) shows an emission shoulder at 533 nm. Data were normalized since the intensity for spectra 1 and 2 were lower for elastin (14,000 arbitrary units of fluorescence, a.u.f.) than spectra 3 and 4 for collagen (a.u.f. 60,000). Images E-G and I-K with their corresponding spectra in H and L, respectively, are from null hearts. In E-G, the collagen is very thin and less structured than in controls (A-C). As shown in H, the emission spectra for collagen (a.u.f. 40,000) and elastin are similar to controls. Significant heterogeneity in collagen and elastin structure was also observed in null hearts (I-K). As shown in L, some cases of significant displacement in collagen structure were observed at 540 nm, along with a broader shoulder on elastin at 520-530 nm.

A focused look at Hif-1a targets revealed downregulation of Hif-1a resulted in downregulation of genes encoding collagen prolyl (P4ha1, P4ha2) and lysyl (Plod2) hydrolyases that are essential for collagen hydroxylation and subsequent folding [32] as well as trimerization PPIases (Ppid, Ppil3, Ppif), and lysyl oxidase (Loxl2) that crosslinks collagen and elastin. In contrast, transcripts for collagen genes, fibronectin, Mmps and their inhibitors (Timps) as well as collagen receptors (Ddr1, Ddr2) were the same for Hltf null hearts as controls. The hydroxyproline assay for total collagen indicated there was no difference (p=0.07) in the total amount of collagen in hearts from null and control mice. However, picrosirius red-staining together with DIC (polarized) microscopy, a method for differentiating collagen and elastic fibers [33,34], revealed disorganization of the collagen fibrillar network in hearts of null mice (Figure 5). Because transcription of genes for collagen biogenesis is downregulated, there is an abundance of single collagen fibrils that are not trimerized, and clumps of collagen that are not crosslinked. Abnormal organization of interstitial and perivascular collagen is evident in hearts of Hltf null mice (Figure 6). Spectral imaging microscopy was used to distinguish elastin (520 nm emission) from collagen (532 nm emission) fibers in control hearts, and to further authenticate alterations in the structure of the collagen network of the Hltf null myocardium (Figure 7). 

**Figure 8:**
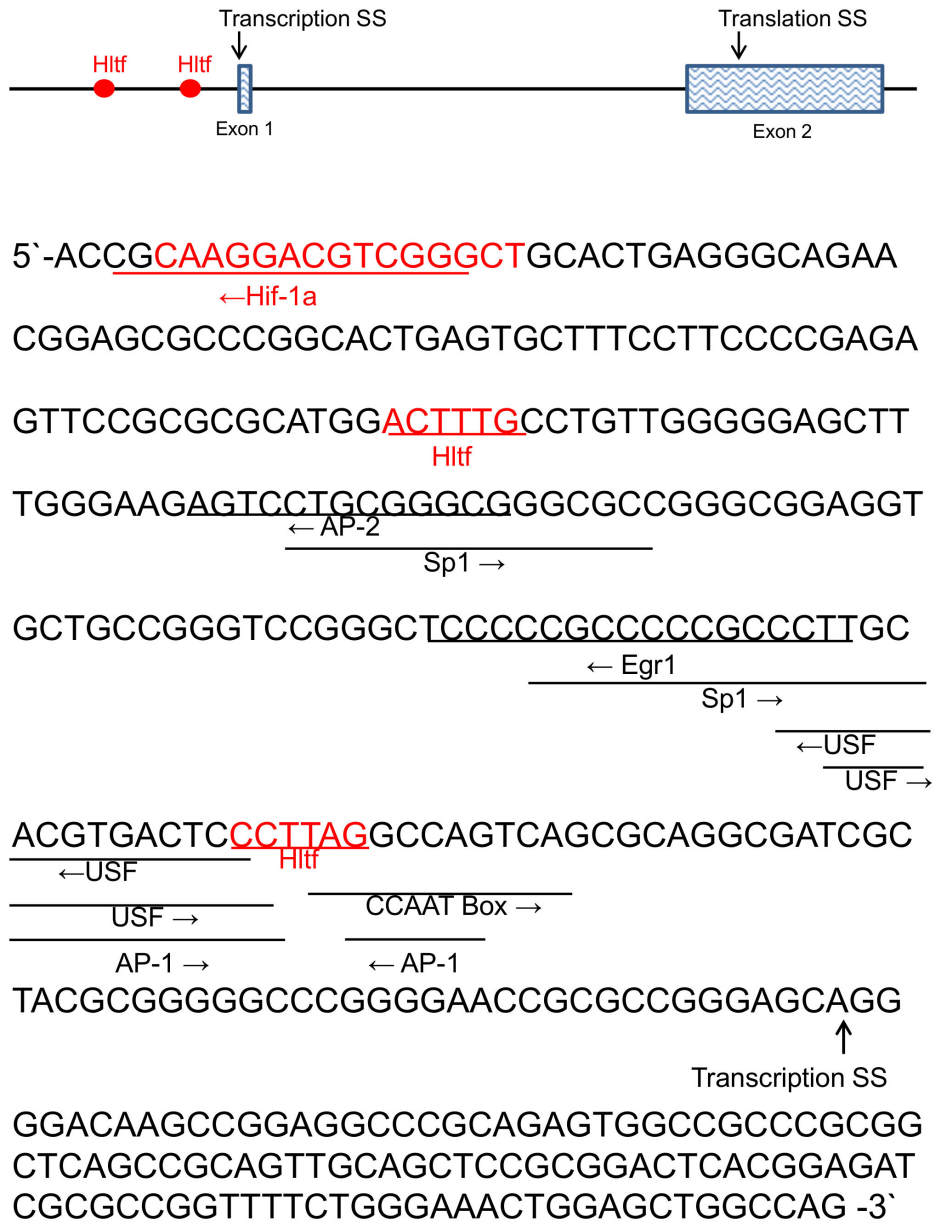
The regulatory region of the Gata4 gene. Transcriptional regulation of Gata4 occurs at a 0.25 kb region upstream of the transcription start site (SS) [35]. Authentic regulatory sites, and putative Hltf binding sites (red) are underlined. Hltf binding to this region of the promoter was authenticated by ChIP-PCR (Figure 2, Panel B). Downregulation of Hif-1a protein binding (red) is predicted from reduced transcription of the Hif-1a gene in Hltf null heart.

**Figure 9:**
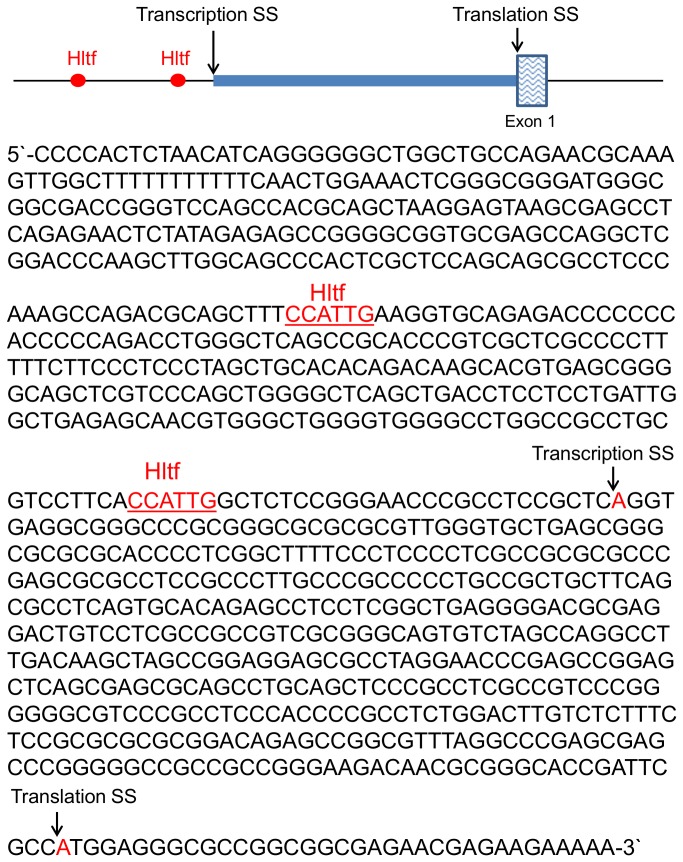
The putative promoter of Hif-1a. Transcription and translation start sites (SS) from mm9 reference genome and NCBI reference sequence NM_010431.2. Putative Hltf binding sites (red) are underlined. Hltf binding to this regulatory region was authenticated by ChIP-PCR (Figure 2, Panel C).

These studies provide the first opportunity to explore primary and secondary regulatory effects of Hltf silencing. First, the 250-bp region in the Gata4 promoter [35] that is important in regulating gene expression in cardiac myocytes is depicted in Figure 8. All previously characterized transcription factor binding sites, and two putative Hltf binding sites, are shown. With the exception of Hif-1a, Hltf, and Nfya, an essential subunit of Nfy, transcript levels for all of the known binding factors are unchanged between Hltf null hearts and controls. ChIP-PCR confirmed Hltf is recruited to the 250-bp regulatory region of the transcriptionally active Gata4 promoter (Figure 2B, lane 1) thereby making Hltf a primary transcriptional activator of Gata4. Second, the -499/+100 bp region of the putative mouse Hif-1a promoter [Chr12, 75008393.75008992 from the eukaryotic promoter database (EPD)] is depicted in Figure 9. Two putative Hltf binding sites are shown. ChIP-PCR confirmed Hltf is recruited to this 599-bp regulatory region of the transcriptionally active Hif-1a promoter (Figure 2C) thereby making Hltf a secondary regulator of Gata4 via its directed transcriptional regulation of Hif-1a.

**Figure 10:**
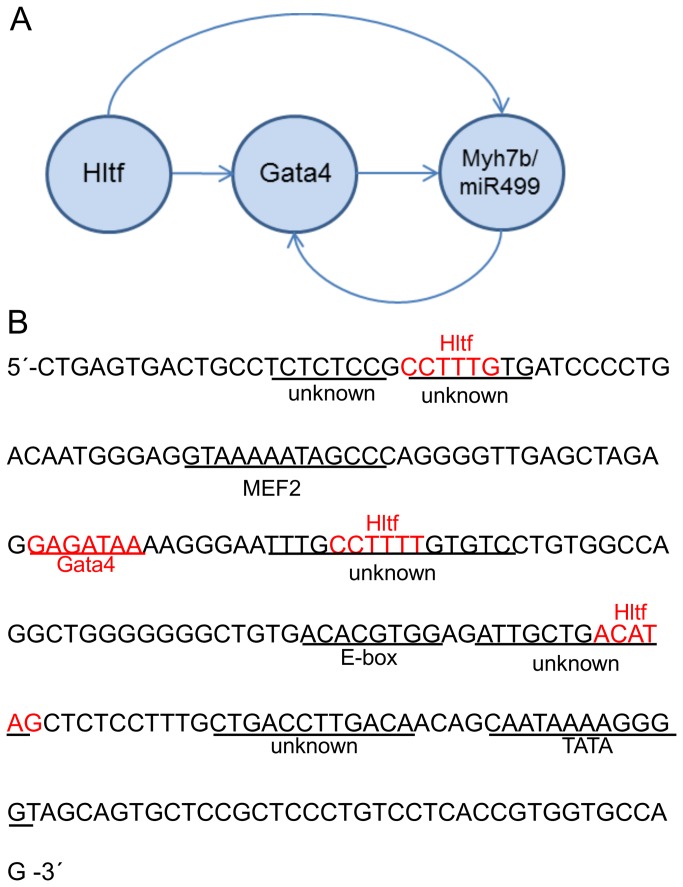
Concurrent transcriptional regulation of Myh7b/miR499 by Hltf. Diagram showing Hltf-regulatory routes (A). Sequence of the Myh7b/miR499 promoter shows authentic transcription factor binding sites (B). Putative Hltf binding elements (red) are three of five highly conserved binding sites previously described as ‘unknown’ [37]. Hltf binding to this shared promoter was authenticated by ChIP-PCR (Figure 2, Panel B). The authentic Gata4 binding site (red) is indicated as Hltf regulates transcriptional availability of Gata4.

Hltf deletion also results in reduced expression of Myh7b/miR499. A common promoter (Figure 10) and co-transcriptional splicing of exon 7 regulate the myosin host gene Myh7b and its intronic miR499, which in turn regulates Gata4 [36]. Quantification of exon 7 expression levels by competitive RT-PCR analysis (Figure 2D) showed the relative amount of exon 7 amplicons included to excluded was the same in both Hltf null and control hearts, as well as brains. These findings emphasize the importance of regulation at the promoter where Gata4, Met2, E-box-binding factors, and five conserved elements (unknowns 1-5) control Myh7b/miR499 expression [37]. Transcript levels for Mef2, as well as factors that bind the E-box [Clock-Bmal1 (Arntl), c-myc, MyoD, E47 (Tcf3), and MyoG] were unchanged between Hltf null hearts and controls. As shown in Figure 10, three of the five highly conserved ‘unknown’ elements [37] are putative Hltf binding sites. ChIP-PCR confirmed Hltf is recruited to the 228-bp regulatory region of the transcriptionally active Myh7b/miR499 promoter (Figure 2B, lane 2). This is further evidence of Hltf’s secondary regulation of Gata4, this time via its direct transcriptional regulation of Myh7b/miR499.

## Discussion

**Figure 11:**
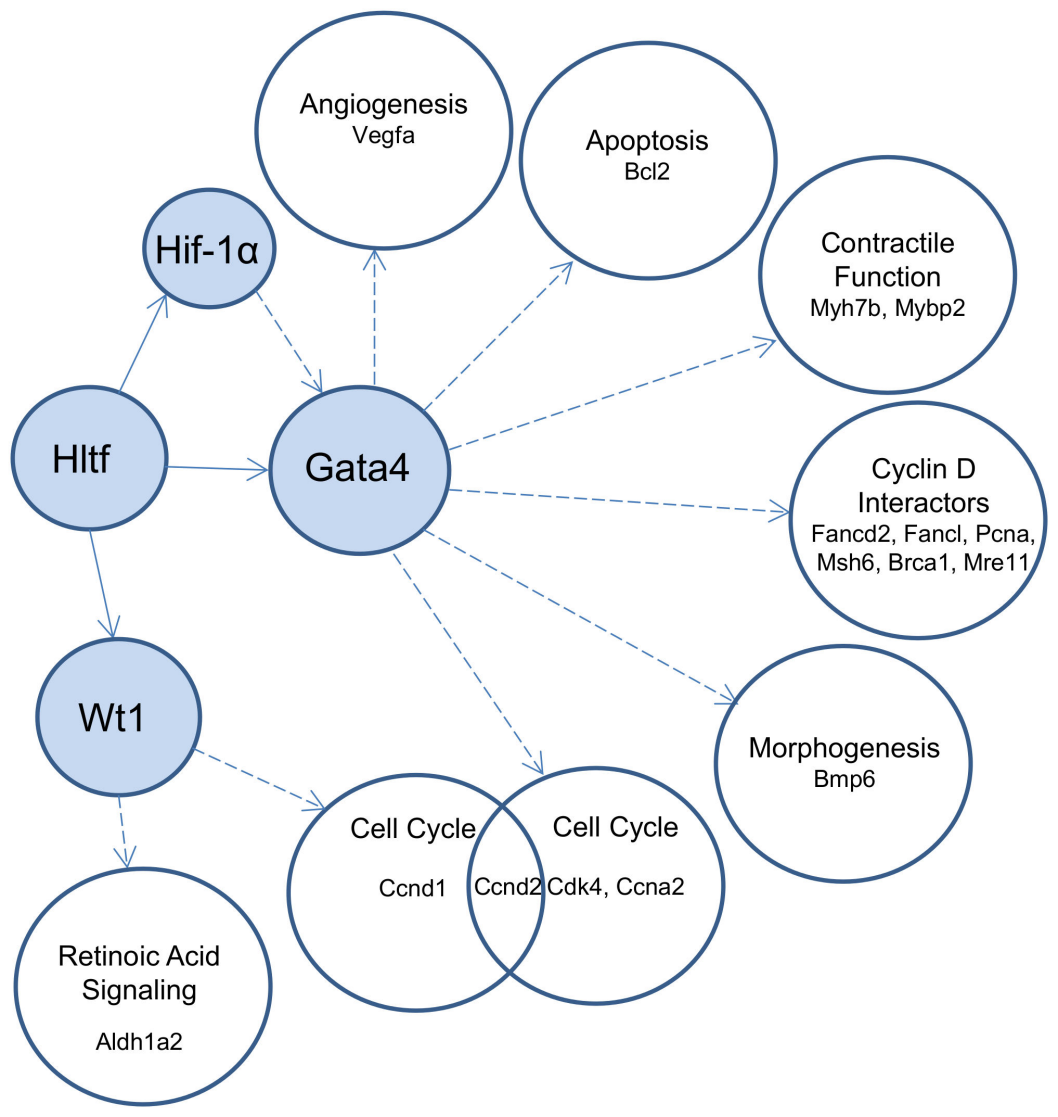
Hltf regulates Gata4-dependent and Gata4-independent pathways. Hltf silencing resulted in the downregulation (threshold 1.1, p < 0.05) of previously unknown target genes (blue circles) and their direct targets (open circles).

 These studies extend our understanding of Hltf’s regulatory role in the maintenance of genomic stability in brain [27] to include maintenance of genomic stability in heart. Hltf controls the expression of key components of the G2/M transition as well as the Fanconi Anemia (FA)/Brca pathway in DNA repair, and cell cycle checkpoint. The hierarchical regulatory relationship of Hltf to Wt1/Gata4/Hif-1a (Figure 11) makes Hltf necessary for heart development and function. Disorganization of the collagen fibrillar network is likely responsible for the semilethal phenotype of newborn Hltf null mice. A role for Hltf in the control of Myh7b/miR499 transcription indicates Hltf participates in the selection of myocardial muscle fiber type. It is also the first demonstration that Hltf has a regulatory role in miRNA-signaling. 

HLTF/Hltf is a prototypical model for alternative splicing. Most human tissues express two HLTF transcripts from alternative splicing of intron 25 in the 3′-UTR [25,38]. Two protein variants are expressed from the same open reading frame, and differ only in their translation start sites (HLTF Met1 and HLTF Met123). Rabbit endometrium expresses two Hltf (alias RUSH) transcripts as a result of hormone-dependent alternative splicing of a 57-bp insert [11]. The full-length α isoform encodes a 1005 amino acid protein. The β isoform results from the inclusion of a 57-bp insert that prematurely truncates the protein at 836 amino acids. Mouse tissues express two Hltf transcripts. Isoform 1, the full-length splice variant, contains exons 1-25. Isoform 2, the truncated splice variant is comprised of exons 1-21 with exon 21 extended via a partial intron retention event. Recently, Capouillez et al [39] observed six splice variants in HeLa cells, and AceView supports the existence of at least six splice variants in mouse. As a result, we previously used a state-of-the-art approach to quantifying alternative splicing of Hltf in control brain [27], an organ with a high amount/rate of alternative splicing. In this study, the same analysis was extended to control heart. In brain [27] the ratio of full-length to truncated Hltf is 5:1, compared with a ratio of 26:1 in heart. All additional splicing events were very low frequency (< 1 event/sample) exon-skip events in both organs. In brain, Hltf proteins from both isoforms were detectable by Western blot in controls [27]. In heart, only the translation product from the full-length isoform was detected. Thus there appears to be a tissue-specific tripartite regulatory mechanism for Hltf that includes promoter usage coupled to alternative splicing and NMD to achieve tissue type-specific expression. Finding such dramatic splicing differences supports the newest views that alternative splicing is a robust component of evolution [40,41]. It also explains why epigenetic silencing is the most efficient way to silence Hltf gene expression. 

Hltf is a phosphonuclear protein [28,29] engaged in the regulation of gene transcription [13,15,16,18], ATP hydrolysis-dependent protein remodeling [10,20-24] and the maintenance of genome stability [27,42-49]. Consistent with the fact that epigenetic silencing of Hltf induces genomic instability, Hltf deletion results in disregulation of the G2/M transition of the cell cycle - with an emphasis on decreased transcript availability of major components in chromosome cohesion and condensation - in heart and brain. Both organs show evidence of increased apoptosis. Moreover, in the Hltf null heart, reduced transcript expression for aurora A kinase and γ-tubulin, plus Brca1 and others members of BASC, is consistent with impaired centrosome maturation, and weakened G2/M checkpoints [50]. Hltf’s regulation of transcript availability of FA family members (a, b, d2, g, i, l) especially the Fancd2/Fanci members of the ID complex, as well as transcripts for the Brca1/Rad51 components of the target Brca1/Brca2/Rad51-complex indicates Hltf signaling is required for the FA/Brca pathway in DNA repair and cell cycle checkpoint [50]. Defects in the FA/BRCA pathway are associated with colorectal, ovarian and breast cancer; and, have potential in therapeutic exploitation [51]. Collectively, these findings indicate silencing Hltf disallows the repair of damaged DNA prior to mitosis, causes defects in DNA damage checkpoint activation, and obstructs DNA double-stranded break (DSB) repair through compromised homologous recombination (HR) between sister chromatids. The end result is cell death via apoptosis in developing heart and brain.

As summarized in Figure 11, Hltf is a direct transcriptional regulator of Gata4, Wt1, and Hif-1a. Downregulation of Gata4 and Wt1 results in concomitant downregulation of cell cycle genes, as well as genes that regulate angiogenesis and contractile function. Hltf is essential for formation of the PE through its regulation of Gata4 and Wt1. Although the inductive signals that initiate formation of the PE are not well characterized, synergy between Gata4 and Wt1 proteins is mandatory [7-9]. Wt1 is required for PE development [52,53]. Downregulation of transcripts for both Wt1 and its gene target Aldh1a2, a rate-limiting enzyme in retinoic acid biosynthesis, indicates changes in epicardial cell survival [54,55]. Also, the timing of smooth muscle cell differentiation that normally occurs after migration of the EPDC into the myocardial wall must be altered. The loss of Gata4 prevents PE outgrowth. Hltf signaling is necessary for formation of the fibrous ECM via Hif-1a, which is required for procollagen secretion and cleavage prior to formation of mature fibrillar collagen under hypoxic conditions [56] that are essential for normal heart development [57]. Given the fact that development and cancer share common molecular pathways, the effects of Hltf-silencing on collagen biosynthesis also helps explain how silencing HLTF promotes tumorigenesis.

Transcription of the glucose transporters Glut1 and Glut9 is downregulated in Hltf null heart and brain [27], respectively. Regardless of the regulatory route - indirect (via Hif-1a) or direct – Hltf is required for the transcriptional activation of genes whose protein products regulate glucose flux. Glut1 and 9 are expressed in vascular smooth muscle cells [58]. Glut1 expression correlates with alterations in cardiac glucose demands during normal cardiogenesis [59], and increases in response to acute hypoglycemia [60]. These data support the hypothesis that the Hltf null myocyte’s reduced capacity to increase glucose transport in response to the demand for ATP contributes to heart failure. 

MiRNA genes are either independent transcriptional units or embedded in introns of host genes. One promoter [36] and co-transcriptional splicing [61,62] regulate the protein-gene/MiRNA-gene unit, Myh7b/miR499. Exclusion of Exon 7 introduces a premature termination codon subjecting Myh7b mRNA - that encodes the major slow-twitch type I myosin isoform - to nonsense mediated-decay, while retaining miR499. The splicing machinery promotes miR499 expression in a tissue where Myh7b is not required. In disagreement with Bell et al [61], who showed only exon 7 exclusion events in heart, we provide evidence for exon 7 inclusion/exclusion events in heart and brain. In heart, where both gene products are important, Hltf is a major regulator at the promoter. Future experimentation is required to validate individual Hltf sites in the Myh7b/miR499 gene promoter. However, the idea that Hltf regulation in heart development is evolutionarily conserved is supported by the presence of all three putative Hltf binding domains in *Xenopus*, chick and mouse [37]. 

In conclusion, genome-wide transcriptome profiling of neonatal (6-8 hour *postpartum*) heart provides conclusive evidence that the functional impact of Hltf silencing derives from defects in the G2/M transition. These findings are consistent with our previous report on the Hltf null brain [27], and with Hltf’s role in promoting genetic stability. In developing heart, Hltf’s regulation of Hif-1a is essential for collagen biogenesis. Disorganization of the collagen fibrillar network may be responsible for the death of neonatal Hltf null mice.

## Supporting Information

Figure S1 **Blood glucose levels for Hltf knockout and control mice.** Values (mean +/- SEM) compared with the Mann-Whitney U-test are significantly different (*p*=0.0084).
(TIF)

Table S1 **DNAnexus alternative splicing analyses quantified the usage of each exon by mapping read counts to the splicesome.** Mapping coordinates from the splicesome were converted to mapping coordinates to the genome. Mappings of the same read to the same genomic location were combined, and their posterior probabilities summed. Exon quantification was performed to show the relative expression of all known Hltf exons in heart.
(XLSX)

Table S2 **DNAnexus alternative splicing analyses quantified the usage for each possible splice junction in control RNA-seq samples.** This resulted in the comparison of different known splice products in addition to the identification of new splice products shown here as Hltf exon-skip events in heart.
(XLSX)

Table S3 **RPKM values were used to identify differentially expressed genes.** Genes whose expression was decreased in Hltf null mouse heart compared with controls are identified here.
(XLSX)

Table S4 **RPKM values were used to identify differentially expressed genes.** Genes whose expression was increased in Hltf null mouse heart compared with controls are identified here.
(XLSX)

Video S1 **Coronary artery fistula in one Hltf knockout (KO) embryo visualized by VisualSonic cardiac echocardiography.**
(MP4)
