# Supplementary figures and images for: Helicase-Like Transcription Factor (Hltf) Regulates G2/M Transition, Wt1/Gata4/Hif-1a Cardiac Transcription Networks, and Collagen Biogenesis
Source: PLoS One. 2013 Nov 20;8(11):e80461. doi: 10.1371/journal.pone.0080461 (PMC3835564; doi:10.1371/journal.pone.0080461)

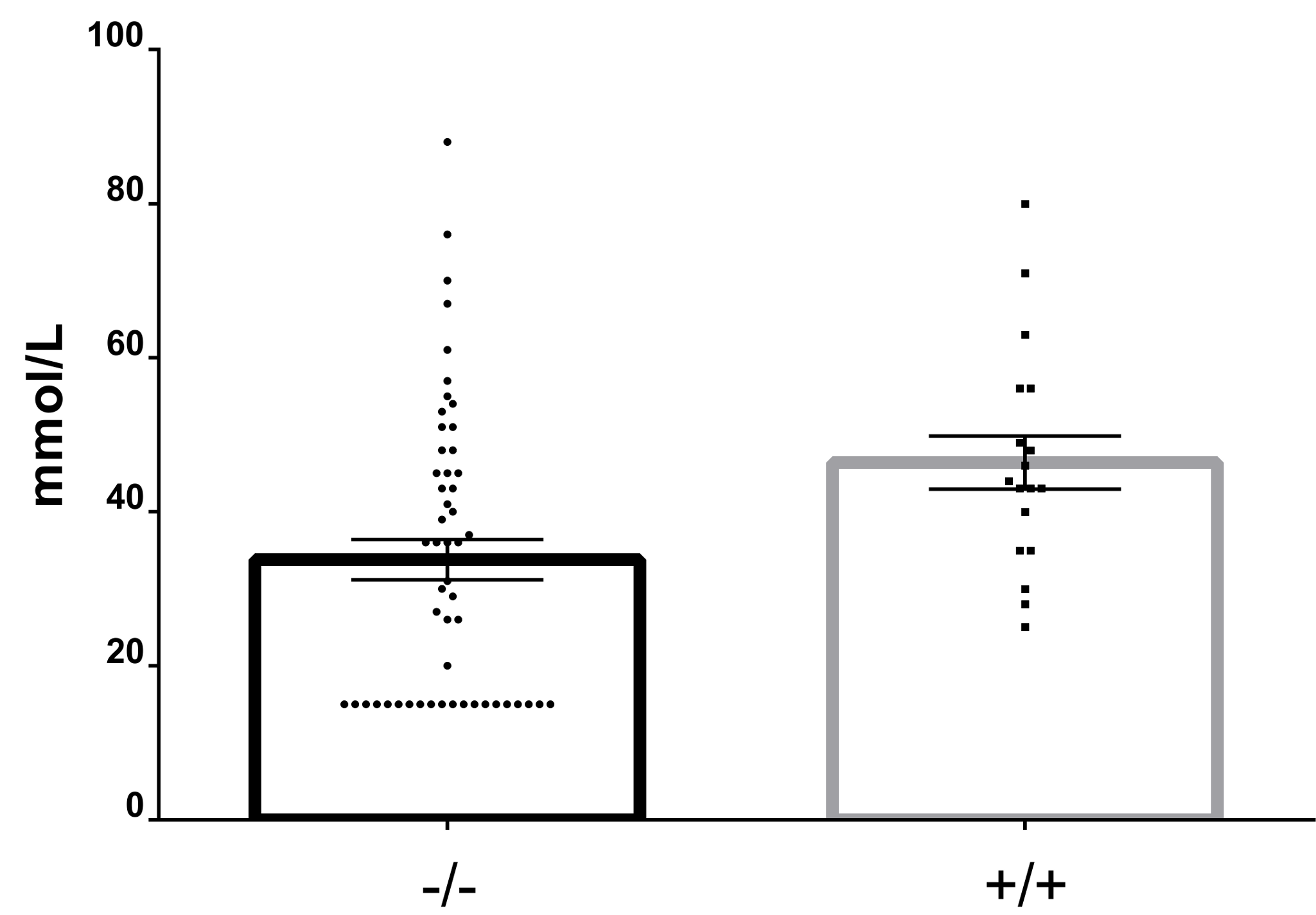

Supplement: Figure S1 — Blood glucose levels for Hltf knockout and control mice. Values (mean +/- SEM) compared with the Mann-Whitney U-test are significantly different (p=0.0084). (TIF) [file pone.0080461.s001.tif]
